# Factors associated with phenotypes of dyspnea in post-COVID-19 condition: a cross-sectional study

**DOI:** 10.1038/s41598-024-64370-4

**Published:** 2024-06-11

**Authors:** Maeve P. Smith, Heather Sharpe, Ronald W. Damant, Giovanni Ferrara, Rachel K. Lim, Michael K. Stickland, Grace Y. Lam

**Affiliations:** 1 Division of Pulmonary Medicine, Department of Medicine, University of Alberta and Alberta Health Services, 3-111C Clinical Sciences Building, 11302 83 Ave NW, Edmonton, T6G 2G3 AB Canada; 2 Alberta Respiratory Centre, University of Alberta, Edmonton, AB Canada; 3 Division of Respiratory Medicine, Cumming School of Medicine, University of Calgary, Calgary, AB Canada; 4 Women and Children’s Health Research Institute, University of Alberta, Edmonton, AB Canada

**Keywords:** Medical research, Signs and symptoms

## Abstract

Post-COVID-19 condition (PCC) is defined as the persistence of symptoms, like fatigue and dyspnea, at least 3 months post-COVID infection. As dyspnea is a common symptom, we attempted to further clinically phenotype those with PCC-associated dyspnea. 1642 adults (average age of 49.6y with 63% female-predominance and BMI of 31.2 kg/m^2^) with physician confirmed diagnosis of PCC from June 2020–April 2023 in Alberta, Canada were included. Those with dyspnea were more likely to be female (56.5%, *p* = 0.005) and have higher BMI (31.3 kg/m^2^ vs. 29.5 kg/m^2^; *p* = 0.0008), history of asthma (21.1% vs. 12.3%; *p* < 0.001), more persistent PCC symptoms (*p* = 0.0001), more functional limitations, as well as lower quality of life (*p* < 0.0001). Multivariable-adjusted logistic regression analysis demonstrated dyspnea was independently associated with fatigue (OR = 4.20; CI = 2.71,6.59) and inversely associated with hospitalization for COVID-19 (OR = 0.53; CI = 0.32,0.91), age (OR = 0.98 per one year of age; CI = 0.96,0.99) and 6-min-walk-distance per 10 m difference (OR = 0.98, CI = 0.96,1.0). Fatigue was a predictor of dyspnea, and was associated with milder infection, higher BMI, and reduced 6-min-walk-distance despite normal pulmonary function. Reduced TLC or DLCO was associated with more severe infection and reduced 6-min-walk-distance. Thus, we speculate there are at least two dyspnea-associated phenotypes: phenotype with pronounced fatigue (normal PFT) and phenotype with pronounced pulmonary abnormalities (abnormal PFT). Improved understanding of the dyspnea-associated phenotypes may allow for better targeted rehabilitation.

## Introduction

Approximately 10–30% of patients that contract Coronavirus disease-19 (COVID-19) continue to experience symptoms lasting ≥ 12 weeks post-infection, termed “Long COVID” or post-COVID-19 condition (PCC)^1^. Individuals who experience PCC often have multi-system symptoms, such as fatigue, dyspnea, headache, sleep disturbances, worsening mental health, joint/chest pain, and brain fog, that may contribute to sub-optimal quality of life^1–3^. Fatigue and dyspnea are the most prevalent PCC symptoms, most notable 60 and > 90 days post-infection^4^.

PCC-related dyspnea is associated with worse sleep, mood, and health-related quality of life, compared to those without dyspnea, adversely affecting activities of daily living^5^. However, persistent dyspnea is not always associated with cardiopulmonary impairment, pulmonary function abnormalities or altered cardiopulmonary response to exercise/exercise intolerance, suggesting that there may be extrapulmonary causes of dyspnea^5–8^. Consequently, we hypothesize that PCC-related dyspnea is a heterogenous symptom that may be due to pulmonary (associated with pulmonary function abnormalities) and extrapulmonary causes (associated with normal lung function). The primary objective of this study is to identify factors that are independently associated with this symptom.

## Method

To gain deeper insights into the nature of PCC-related dyspnea, we undertook a cross-sectional study of all patients (1642 adult patients, ≥ 18 years) who received care from June 2020–April 2023 in any of the publicly funded post-COVID clinics across Alberta, Canada (Interprofessional Outpatient Long COVID clinics at the Kaye Edmonton Clinic, Peter Lougheed Centre and Rockyview Hospital as well as the Peter Lougheed Centre Post-COVID Respiratory Clinic) were included in the study. Confirmed infection was determined by either polymerase chain reaction (PCR), patient-reported rapid antigen test (RAT) positivity (*n* = 1544), or strong clinical suspicion of COVID-19 infection as determined by two separate clinicians (*n* = 98). Of the 1642 patients who received care for PCC in one of the participating sites, data on self-reported dyspnea (binary outcome) was available for 1553 patients (94.6%). Due to variability in staffing resource at various clinics, pulmonary function test (PFT) and 6-min walk distance (6MWD) data were only recorded for 959 (62%) subjects.

PCC was defined in accordance with the World Health Organization (WHO): a confirmed or suspected history of SARS-CoV-2 infection; symptoms that cannot be explained by another diagnosis; and symptoms that last for at least 3 months from the onset of the infection^9^. Patients were referred to the PCC clinic either by their family physicians or at discharge following a COVID-19 pneumonia hospital admission. All patients seen at the various sites who met the definition of PCC were included in this study without exclusion. Patients with PCC were divided into two cohorts: those reporting subjective symptoms of exertional dyspnea at the time of initial consultation or those who did not (ie: binary outcome). Demographic data (including age, sex, body mass index (BMI)), clinical data (including PCC symptoms, previous medical history, hospitalization history, pulmonary function, and six-minute walk test (6MWT)^10^) were collected. Self-reported data included questionnaires relating to functional status (as measured by the Post-COVID-19 Functional Status Scale (PCFS)^11^), health-related quality of life (as measured by the Eq. 5D5L-vas)^12^, symptoms of dyspnea (as measured by medical research council (MRC) dyspnea scale), self-reported fatigue, depression (as measured by the Patient Health Questionnaire (PHQ-9)^13^, and anxiety (as measured by the General Anxiety Disroder-7 (GAD-7)^14^). All measurement tools were analyzed per published cutoffs as referenced. All data was collected at initial consultation an average 8 months’ post-infection.

Multivariate logistic regression analyses were conducted using sex, age, BMI, hospitalizations, symptoms of fatigue, forced expiratory volume in one second (FEV_1_), forced vital capacity (FVC), total lung capacity (TLC), diffusing capacity of the lungs for carbon monoxide (DLCO), 6-min walk distance (6MWD) and exertional desaturation determined prior to the start of the study as independent confounders of dyspnea and fatigue. The correct Akaike Information Criterion (AICc) and area under the curve (AUC) for the dyspnea model is 545.5 and 0.79 respectively. The AICc and AUC for the fatigue model is 718.5 and 0.75 respectively.

Multivariate linear regression analysis was conducted with the same list of confounders to determine independent covariates that are associated with reduced TLC and DLCO. Statistical analyses were done with Prism 9.0 using non-parametric T-tests, Fisher’s exact test and multivariate regression analyses. β-coefficients or odds ratios with 95% confidence intervals (CIs) and *p*-values were reported. Statistical significance was considered for *p*-value < 0.05.

This study was approved by and conducted in accordance with the University of Alberta Research Ethics Board (Pro00104564) with a waiver of individual consent given that de-identified data was accessed for this study.

## Results

1553 individuals with PCC included in the study with a baseline average age of 49.7y, female-predominance (63%) and mean BMI of 30.9 kg/m^2^. Of these, 1164 (75.0%) indicated that they experienced dyspnea at initial consultation, while 389 (25.0%) did not (Table 1). Baseline exploratory analyses demonstrated that those with dyspnea were more likely to be female (56.5%, *p* = 0.005), had a higher BMI (31.3 kg/m^2^ versus 29.5 kg/m^2^; *p* = 0.0008), were more likely to have a previous history of asthma (21.1% versus 12.3%; *p* < 0.001), and were more removed from the acute infection (264d versus 233d; *p* = 0.0001). There was no statistically significant difference in the history of hospitalizations, ICU admission, pre-existing COPD, or interstitial lung disease diagnosis. Clinically, patients with dyspnea self-reported higher rates of fatigue (85.6% versus 53.3%), gastrointestinal symptoms (32.9% versus 20.0%), and neurocognitive symptoms (67.6% versus 44.7%) (all *p* < 0.0001), with no statistically significant difference in neurologic symptoms. Individuals experiencing dyspnea self-reported greater functional status limitations (median PCFS 3 (CI 3, 3) versus 3 (CI 3, 3)), lower quality of life scores (median Eq. 5D-5L-vas score 50 (CI 50, 50) versus 64 (CI 60, 70)), as well as more depressive (median PHQ-9 score 11 (CI 10, 11) versus 7 (CI 6, 8)) and anxiety (median GAD-7 score 7 (CI 3, 6) versus 5 (CI 6, 7)) symptoms. Functionally, those with dyspnea demonstrated a shorter 6MWD (median 432 m (CI 423, 446) versus 485 m (CI 471, 505)), lower FEV_1_ (median 92% (CI 91, 93) versus 95% (CI 92, 97)), lower FVC (median 95% (CI 93, 95) versus 97% (CI 93, 100)), higher FEV_1_/FVC ratio (median 78.8% (CI 78.2, 79.3) versus 77.8% (CI 76.8, 78.4)), and lower TLC (median 99.0% (CI 97, 101) versus 101.0% (CI 99, 104)). There was no significant difference in O_2_ nadir during the 6MWT, heart rate at completion of 6MWT, or DLCO.

**Table 1:** Baseline Demographics of the study.

	No Dyspnea (*n* = 389)	Dyspnea (*n* = 1164)	*p*-value
Age, years (SD)	51.2 (14.5)	49.1 (13.6)	0.006
Female sex, *n* (%)	220 (56.6)	753 (64.6)	0.005
BMI, kg/m^2^ (SD)	29.5 (6.5)	31.3 (7.7)	0.0008
Time from infection, d (SD)	233 (147)	264 (164)	< 0.0001
Hospitalization, *n* (%)	115 (29.5)	299 (25.7)	NS
ICU, *n* (%)	35 (9.0)	135 (11.6)	NS
Past medical history			
Asthma, *n* (%)	48 (12.3)	246 (21.1)	< 0.0001
COPD, *n* (%)	7 (1.8)	35 (3.0)	NS
Interstitial Lung Disease, *n* (%)	1 (0.3)	15 (1.3)	NS
Pulmonary function test			
FEV1, % (SD)	94.3 (15.3)	90.6 (24.8)	0.02
FVC, % (SD)	96.7 (17.8)	92.4 (26.2)	0.02
FEV1/FVC, % (SD)	76.9 (7.6)	77.8 (8.3)	0.02
TLC, % (SD)	101.7 (17.8)	98.0 (18.8)	0.03
DLCO, % (SD)	87.7 (16.3)	86.9 (18.9)	NS
6-Minute walk test			
6-min walk distance, m (SD)	478.5 (116)	421.3 (130)	< 0.0001
% 6-min walk distance > predicted LLN*	53%	70%	< 0.0001
O2 nadir, % (SD)	91.9 (3.6)	91.4 (4.4)	NS
Heart rate end of test, bpm (SD)	109 (18.5)	109 (16.1)	NS
Associated symptoms			
Fatigue, *n* (%)	212 (53.3)	996 (85.6)	< 0.0001
Gastrointestinal, *n* (%)	76 (20.0)	383 (32.9)	< 0.0001
Neurologic, *n* (%)	245 (63.0)	959 (65.1)	NS
Neurocognitive, *n* (%)	174 (44.7)	787 (67.6)	< 0.0001
Patient reported metrics			
MRC dyspnea, median (IQR)^$^	1 (1; 1–2)	2 (1; 2–3)	< 0.0001
PCFS, median (IQR)^^^	3 (1; 2–3)	3 (1; 3–4)	< 0.0001
Equation 5D5L-vas, median (IQR))	64 (31.25; 49.75–81)	50 (34.75; 35.25–70)	< 0.0001
PHQ9, median (IQR)^@^	7 (10; 2–12)	11 (9.25; 6–15.25)	< 0.0001
GAD7, median (IQR)^#^	5 (8; 1–9)	7 (8; 3–11)	< 0.0001

SD = standard deviation; BMI = body mass index; ICU = intensive care unit; FEV_1_ = (percentage predicted) forced expiratory volume in 1 s; FVC = (percentage predicted) forced vital capacity; TLC = total lung capacity; DLCO = diffusion capacity; bpm = beats per minute; LLN = lower limit of normal; MRC = medical research council, IQR = interquartile range; PCFS = post-covid functional scale; PHQ-9 = patient health questionnaire; GAD-7 = general anxiety disorder-7.

*LLN determined using the Enright & Sherrill equation (Enright et al. 1998. Am J Respir Crit Care Med).

^$^MRC dyspnea interpretation: 1 = not troubled by breathlessness except with strenuous exercise; 2 = troubled by shortness of breath when hurrying on the level of walking up a slight hill; 3 = waslks slower than people of the same age on the level because of breathlessness or has to stop for breath when walking at own pace on the level; 4 = stops for breath after walking about 100 years or after a few minutes on the level; 5 = too breathless to leave the house or breathless when dressing or undressing.

^^^PCFS interpretation: 0 = no limitations in everyday life and no symptoms, pain, depression or anxiety related to the infection; 1 = negligible limitations in everyday life performing all usual duties/activities, although still have persistent symptoms, pain, depression or anxiety; 2 = suffer from limitations in everyday life as occasional need to avoid or reduce usual duties/activities or need to spread these over time due to symptoms, pain, depression or anxiety. Able to perform all activities without any assistance; 3 = suffer from limitations in everyday life as not able to perform all usual duties/activities due to symptoms, pain, depression or anxiety. Able to take care of self without any assistance; 4 = suffer from severe limitations in everyday life: not able to take care of self and therefore dependent on nursing care and/or assistance from another person due to symptoms, pain, depression or anxiety.

^@^PHQ-9 interpretation ranges: 0–4 = none; 5–9 = mild depression; 10–14 = moderate depression; 15–19 = moderately severe depression; 20–27 = severe depression.

^#^GAD7 interpretation ranges: 0–4 = low risk of anxiety; 5–9 = mild risk; 10–14 = moderate risk; 15 +  = severe risk.

Multivariate logistic regression analysis was employed to identify factors that were independently associated with dyspnea, fatigue and multivariate linear regression was used to identify factors associated with reduced TLC and DLCO (Tables 2 and 3). Dyspnea was independently associated with younger age (OR = 0.98; CI = 0.96, 0.99), fatigue (OR = 4.20; CI = 2.71, 6.59), no hospitalization for COVID-19 (OR = 0.53; CI = 0.32, 0.91) and reduced distance walked on 6MWT per 10 m difference (OR = 0.98, CI = 0.96, 1.0). No other relationships were found, notably in lung function metrics. Correspondingly, fatigue was found to be a strong predictor of dyspnea (OR = 4.29; 2.75, 6.73), and associated with never hospitalized status (OR = 0.34; CI = 0.2, 0.59), higher BMI (OR = 1.05); CI = 11,1.09) and reduced 6MWD per 10 m difference (OR = 0.97; CI = 0.94, 0.99). Multivariate linear regression analysis was next performed to identify factors that were independently associated with TLC and DLCO. TLC reduction was associated with male sex (β-estimate = 3.92; CI = 2.93, 4.90), younger age (β-estimate = 0.07, CI = 0.04, 0.10), and other PFT abnormalities (including lower FEV_1_, FVC, and DLCO). DLCO reduction was independently associated with lower BMI (β-estimate = 0.57; CI = 0.42, 0.72), male sex (β-estimate = 3.77; CI = 1.24, 6.31), hospitalizations (β-estimate = − 7.90; CI = − 10.65, − 5.16), lower TLC (β-estimate = 0.32; CI = 0.12, 0.52), lower 6MWD (β-estimate = 0.02; CI = 0.01, 0.03), and O_2_ exertional desaturation (β-estimate = 0.28; CI = 0.02, 0.54).

**Table 2:** Multivariate logistic regression analysis of covariates independently associated with dyspnea, and fatigue.

Co-variates	Odds Ratio (95% confidence interval)	
	Dyspnea	Fatigue
Age	**0.98 (0.96, 0.99)**	0.99 (0.97, 1.00)
Female Sex	0.93 (0.58, 1.49)	1.19 (0.71, 1.97)
BMI	1.03 (1.0, 1.06)	**1.05 (1.01, 1.09)**
Hospitalization	**0.53 (0.32, 0.91)**	**0.34 (0.2, 0.59)**
Fatigue	**4.20 (2.71, 6.59)**	–
Dyspnea	–	**4.29 (2.75, 6.73)**
FEV_1_	0.99 (0.97, 1.01)	1.01 (0.99, 1.03)
FVC	0.97 (0.93, 1.01)	0.97 (0.93, 1.01)
TLC	1.02 (0.99, 1.06)	1.03 (0.98, 1.07)
DLCO	0.99 (0.97, 1.00)	1.01 (1.0, 1.03)
6MWD	**0.98 (0.96, 1.0)**	**0.97 (0.94, 0.99)**
Exertional desaturation	0.97 (0.92, 1.02)	0.96 (0.96, 1.07)

BMI = body mass index; FEV_1_ = (percentage predicted) forced expiratory volume in 1 s; FVC = (percentage predicted) forced vital capacity; TLC = total lung capacity; DLCO = diffusion capacity; 6MWD = 6-min walk distance per 10 m difference.

Significant values are in [bold].

**Table 3:** Multivariate linear regression analysis of covariates independently associated with reduced TLC and DLCO.

Co-variates	β-estimate (95% confidence interval)	
	TLC	DLCO
Age	**0.07 (0.04, 0.10)**	− 0.01 (− 0.09, 0.07)
Female sex	**3.92 (2.93, 4.90)**	**3.77 (1.24, 6.31)**
BMI	0.03 (− 0.04, 0.09)	**0.57 (0.42, 0.72)**
Hospitalization	− 0.80 (− 1.94, 0.35)	− **7.90 (**− **10.65,** − **5.16)**
Fatigue	0.67 (− 0.41, 1.76)	2.21 (− 0.46, 4.87)
Dyspnea	0.55 (− 0.44, 1.53)	− 1.97 (− 4.39, 0.46)
FEV_1_	**0.16 (0.11, 0.21)**	0.03 (− 0.09, 0.14)
FVC	**0.82 (0.77, 0.86)**	0.07 (− 0.13, 0.28)
TLC	–	**0.32 (0.12, 0.52)**
DLCO	**0.05 (0.02, 0.09)**	–
6MWD	0.00 (− 0.00, 0.01)	**0.02 (0.01, 0.03)**
Exertional desaturation	0.04 (− 0.07, 0.14)	**0.28 (0.02, 0.54)**

BMI = Body mass index; FEV_1_ = (percentage predicted) forced expiratory volume in 1 s; FVC = (Percentage predicted) forced vital capacity; TLC = Total Lung Capacity; DLCO = Diffusion capacity; 6MWD = 6-min walk distance.

Significant values are in [bold].

### Ethics approval and consent to participate

The study was approved by the University of Alberta Research Ethics Board (Pro00104564) and conducted in full accordance with the ethics board guidelines.

## Discussion

**Figure 1:**
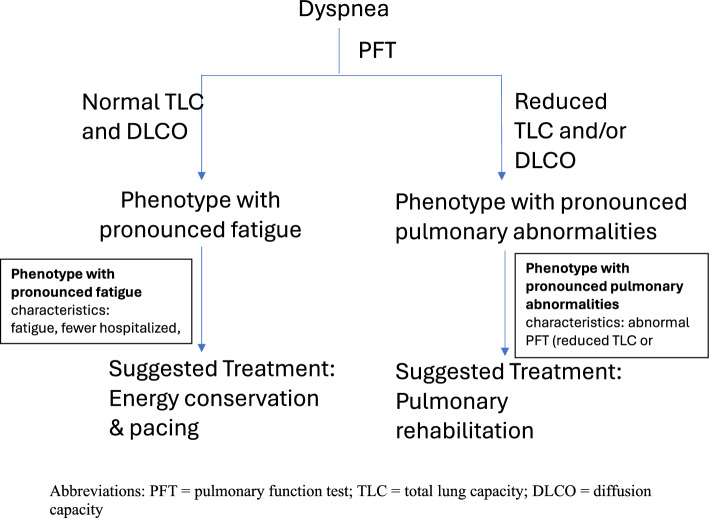
Dyspnea is Associated with Multiple PCC Phenotypes.

Consistent with existing literature, this study identified dyspnea as a common symptom of PCC^15^ which was more likely to impact females^16^. Importantly, we have demonstrated that PCC-related dyspnea was associated with a greater burden of symptoms and poorer health-related quality of life. Elevated BMI and a prior history of asthma were additional independent factors associated with dyspnea development. Self-reported dyspnea in patients with PCC is not always associated with cardiorespiratory abnormalities^5,7,8,17^. Our results indicate that PCC-related dyspnea was not associated with pulmonary functional abnormality or exertional hypoxia but was instead strongly associated with fatigue (and vice versa). However, our data also indicates that the characteristics and associated risk factors for patients with dyspnea do not completely overlap with those endorsing fatigue, though there is likely some patients experiencing both fatigue and dyspnea where dyspnea exists in context of fatigue (Table 2). Surprisingly, reduced TLC or DLCO was not significantly associated with dyspnea or fatigue but was associated with reduced exercise capacity, suggesting those with PFT abnormalities represent a distinctly different but clinically relevant phenotype (ie: more likely associated with hospitalization/severe acute infection; Table 3). Consequently, we speculate that there are at least two distinct dyspnea-related phenotypes (Fig. 1): 1. Phenotype with pronounced fatigue (the predominant phenotype) that is characterized by normal lung function where dyspnea is secondary to fatigue; and 2. Phenotype with pronounced pulmonary abnormalities that is characterized by PFT abnormalities where intrinsic pulmonary defects could result in dyspnea.

Further evidence to support the existence of two distinct dyspnea phenotypes comes from divergent observational trajectory data between those with documented pulmonary abnormalities versus those with fatigue. Observational studies have found that a restrictive pattern with diffusion abnormalities were the predominant findings around 12 weeks post infection, which was associated with varying degrees of improvement over the following year^18,19^. Correspondingly, computed tomography (CT) findings of ground glass or consolidative opacities evolved over months into reticular or fibrotic changes with or without architectural distortion likely mirroring PFT changes^20^. In contrast, patients with PCC-related fatigue report either persistent or increasingly severe fatigue as measured by patient-reported fatigue scores^21^.

It is must be noted that our approach to distinguishing the phenotype with pronounced pulmonary abnormalities from phenotype with pronounced fatigue rests on PFT findings, a modality that reliably has confirmed consistent post-COVID findings around the world^5,7,8^. The reliance on PFTs for our clinical phenotyping was also chosen because it can be performed in rural or remote locations with limited healthcare resources and has been found to correlate well with abnormalities detectable on more costly clinical imaging modalities like SPECT-CT^22^ or research-based testing modalities such as ^129^Xe lung MRI^23^. However, as other more advanced, multimodal imaging techniques become available, pulmonary abnormalities may be identified in patients with PCC despite normal pulmonary function testing. Electrical impedance tomography, a radiation free technique that infers local ventilation based on changes in regional impedance, demonstrated higher regional inhomogeneity in patients with PCC-related dyspnea but otherwise normal PFT at 1-year post-infection^6^. Perhaps minute aberrancy in ventilatory undetectable by conventional testing could explain for PCC-related dyspnea, though it remains unclear how clinically relevant these differences are on a population level. Thus, more work is needed to better understand PCC-related dyspnea which may lead to future phenotypic reclassification.

Dysfunctional breathing can be seen in patients with PCC^24^, the correction of which may universally improve dyspnea regardless of the phenotype. Additionally, accurate classification of PCC phenotypes is critical to allow for the selection of the most appropriate management^15^. As indicated by Beaudry and colleagues^7^ individuals who experience persistent dyspnea without cardiopulmonary impairment may benefit from general rehabilitation strategies that target strengthening or conditioning. Pulmonary rehabilitation (PR) has been shown to improve dyspnea, exercise capacity, lung function, fatigue and 6MWD for individuals with PCC^25,26^. Many patients with PCC-related fatigue also experience post-exertional symptom exacerbation (PESE) who are vulnerable to more intensive rehabilitation. Consequently, these patients would more likely benefit from energy conservation measures, such as paced exertional activities to reduce fatigue^27^ while those with pulmonary defects who may be better managed using conventional PR techniques^28^. Thus, our research may help identify what PR interventions that may be of most use to individuals based on phenotype, and may provide personalized approaches to rehabilitation efforts.

This study involved a cohort of well-characterized patients with physician confirmed PCC representative of the estimated 4.7 residents in the province. However, there are a few limitations to note. First, there may be a selection bias as the population in the study was identified from a specialty clinic, which may represent a more severe PCC sub-group than those receiving care in primary care settings. Second, the clinics are in urban centres, which may result in a selection bias with fewer rural patients, although virtual care options were provided to facilitate access. Third, the lack of a comparator group (such those with a history of COVID-19 infection without PCC) limits the ability to identify risk factors of PCC-associated dyspnea development. However, even with the current cross-sectional approach, we can identify associated features of different dyspnea phenotypes which may provide relevant considerations for treatment selection. Fourth, some outcomes data in this study rely on self-reporting of symptoms which may result in a recall bias. Finally, the tools used for symptom assessment were implemented prior to international validated tools in PCC were published. The future use of the recently published consensus-based core-outcomes measurement tools may improve the validity and generalizability of our data^29^.

Dyspnea is a common presenting symptom of PCC and is associated with a greater burden of symptoms and poorer health-related quality of life. This study suggests the existence of at least two dyspnea phenotypes that may be distinguished using PFTs allowing for a targeted clinical approach to rehabilitation. Those with PFT abnormalities (phenotype with pronounced pulmonary abnormalities) may benefit from PR while those without PFT abnormalities (phenotype with pronounced fatigue) may be more suitable for general pacing-focused rehabilitation interventions.

## Data Availability

The datasets used and analysed during the current study are available from the corresponding author on reasonable request.

## Abbreviations

PCC: Post-COVID condition
PFT: Pulmonary function test
6MWT: 6-Minute walk test
6MWD: 6-Minute walk distance
FEV_1_: Forced expiratory volume in 1 s
FVC: Forced vital capacity
TLC: Total lung capacity
DLCO: Diffusion capacity
